# The Highly Embryogenic *Brassica napus* DH4079 Line Is Recalcitrant to *Agrobacterium*-Mediated Genetic Transformation

**DOI:** 10.3390/plants12102008

**Published:** 2023-05-17

**Authors:** Antonio Calabuig-Serna, Ricardo Mir, Rosa Porcel, Jose M. Seguí-Simarro

**Affiliations:** Cell Biology Group-COMAV Institute, Universitat Politècnica de València, 46022 Valencia, Spain; ancaser3@upv.es (A.C.-S.); roporrol@upv.es (R.P.)

**Keywords:** *Agrobacterium tumefaciens*, *Agrobacterium rhizogenes*, canola, DH4079, in vitro culture, oilseed rape, rapeseed, transgenic

## Abstract

*Brassica napus* is a species of high agronomic interest, used as a model to study different processes, including microspore embryogenesis. The DH4079 and DH12075 lines show high and low embryogenic response, respectively, which makes them ideal to study the basic mechanisms controlling embryogenesis induction. Therefore, the availability of protocols for genetic transformation of these two backgrounds would help to generate tools to better understand this process. There are some reports in the literature showing the stable transformation of DH12075. However, no equivalent studies in DH4079 have been reported to date. We explored the ability of DH4079 plants to be genetically transformed. As a reference to compare with, we used the same protocols to transform DH12075. We used three different protocols previously reported as successful for *B. napus* stable transformation with *Agrobacterium tumefaciens* and analyzed the response of plants. Whereas DH12075 plants responded to genetic transformation, DH4079 plants were completely recalcitrant, not producing any single regenerant out of the 1784 explants transformed and cultured. Additionally, an *Agrobacterium rhizogenes* transient transformation assay was performed on both lines, and only DH12075, but no DH4079 seedlings, responded to *A. rhizogenes* infection. Therefore, we propose that the DH4079 line is recalcitrant to *Agrobacterium*-mediated transformation.

## 1. Introduction

Genetic transformation consists of the introduction of a foreign DNA molecule into a genome, generating a genetically modified organism. The first reports on plant genetic transformation date from 1983 and used *Agrobacterium tumefaciens* as a transformation vector to introduce foreign DNA into the genomes of different plant species [1]. Among the different technologies available for plant genetic transformation, *Agrobacterium*-mediated transformation is nowadays the most used approach. The use of genetically modified crops has increased both plant productivity and farmer profits, while reducing the use of pesticides, among other advantages [2]. Indeed, a total of 438 genetically modified plant events have been approved worldwide [3]. More recently, the impact of biotechnology on agriculture has been enhanced by different genome editing techniques, such as zinc-finger nucleases, transcription activator-like effector nucleases (TALENs), and clustered regularly interspaced short palindromic repeats (CRISPR) and CRISPR-associated protein 9 (Cas9). The use of CRISPR/Cas9 technology for genome editing implies the previous generation of genetically modified plants [4]. On the other hand, the use of genetic transformation to generate gain and loss-of-function lines in different model and crop species has helped us to understand gene function and constitutes a powerful biotechnological tool for basic and applied plant genetic and molecular research [5]. Therefore, the availability of efficient plant transformation protocols for different crops and plant species is crucial for the improvement of crop performance for agriculture, as well as for the generation of new biotechnological research tools.

Microspore embryogenesis is a process whereby the male gametophyte deviates from its original developmental pathway and is induced to develop as a haploid embryo whose genome can then be doubled to become a doubled haploid (DH), fully homozygous individual [6]. This process constitutes a powerful biotechnological tool for both basic research [7,8] and applied plant breeding [9]. *Brassica napus* is a model species for the study of this experimental pathway [10,11] and the processes involved in its in vitro induction [12,13,14,15,16]. This is due to the high potential of the microspores of some genotypes, such as the DH4079 line, to become induced to embryogenesis [11,17]. In parallel, other lines such as the DH12075 line, consistently show a low response to the same embryogenesis-inductive conditions [17]. Thus, *B. napus* is an interesting target species to use to combine transformation and microspore embryogenesis protocols in order to develop DH individuals from haploid-transformed microspores, thereby fixing the transgene in homozygosis and avoiding the occurrence of hemizygous regenerants [11]. The development of successful transformation protocols for *B. napus* lines with different embryogenic competence would greatly help to develop biotechnological tools to study the cellular and molecular basis of this morphogenic process.

The type of explant tissue, the selection marker used and the genotype have been reported among the main factors affecting the efficiency of stable *B. napus* transformation [18,19]. Some of the first attempts to transform *B. napus* were based on the use of cotyledon petioles as explants [20]. Afterwards, other effective protocols have been developed, also based on the use of cotyledons [21] or other explants such as stem portions [22], protoplasts [23] or hypocotyl sections [24]. Among the different selection markers available, kanamycin has been commonly used as an effective selection agent in *B. napus* transformation, whereas the use of BASTA has been less frequent [25]. The third major factor is the genotype. In other members of the brassicaceae family, such as *B. oleracea*, there are protocols to stably transform with *Agrobacterium* different genotypes of crops such as cauliflower and broccoli, among others, with low genotype dependence [26]. However, different *B. napus* genotypes show different responses to *Agrobacterium*-mediated transformation [24]. Several works reported the successful stable transformation of the low embryogenesis-responsive lines Westar [20,22] and DH12075 [27,28], whereas to our knowledge, no transgenic lines have been described so far for the highly embryogenic DH4079 line. Whether DH4079 is extremely recalcitrant to *Agrobacterium*-mediated transformation, or simply no transformed DH4079 lines have been reported yet, is unknown.

In this work, we aimed to determine an optimal protocol for *Agrobacterium*-mediated stable transformation of the *B. napus* DH4079 line using different conditions, including different plant explants, plasmids, *Agrobacterium* strains and incubation times. As references to compare with, we also attempted stable transformation of the DH12075 line with the same protocols and analyzed transient transformation mediated by *Agrobacterium rhizogenes* in DH4079 and DH12075 seedlings. Our results point to a genetic recalcitrance of the DH4079 line but not of the DH12075 line, which was possible to transform with both *Agrobacterium* species. These results make these *B. napus* lines convenient models to study the mechanisms of recalcitrance to *Agrobacterium*-mediated transformation in plants.

## 2. Results and Discussion

**Figure 1:**
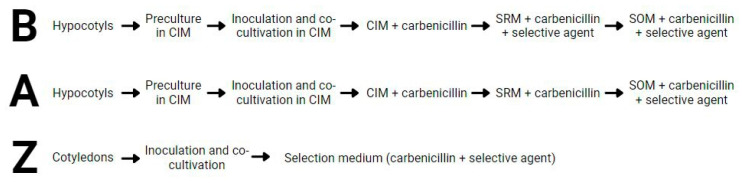
Scheme of the different steps of the three transformation protocols (B, A and Z) used in this work. See text for further details.

In this work, we used three protocols for stable transformation and regeneration (namely B, Z and A, Figure 1) previously described for *B. napus* with some modifications [21,24]. These protocols were assayed using the YC3.6-Bar, PM-YC3.6-LTI6b and pCRISPR plasmids.

Hypocotyl and cotyledon explants were excised from *B. napus* donor plants (Table 1). In total, we excised and cultured 1784 explants of the DH4079 line and 1077 explants of the DH12075 line, the latter of which was used as a reference (control) of a previously transformed *B. napus* line [27,28]. Each protocol was tested in the two *B. napus* lines, transforming them with different plasmids depending on the case. We first assessed the efficiency of plant regeneration of the three protocols used in this study (Table 1, “Plants/explant”), and then the number of regenerated explants successfully transformed (Table 1, “Positive plants/explant”). We found that the genotype and the plasmid type had no significant effect on the rate of plant regeneration, since none or very few regenerants were obtained from both lines and transformation events using protocols B and Z. However, all lines and transformation events produced regenerants with protocol A. Protocol A was found to be significantly better than protocol B in terms of plant regeneration, estimated as the number of regenerated plants per explant (Table 2). The reasons for such better performance are discussed next.

### 2.1. The Selective Agents Have a Critical Role in Plant Regeneration

**Figure 2:**
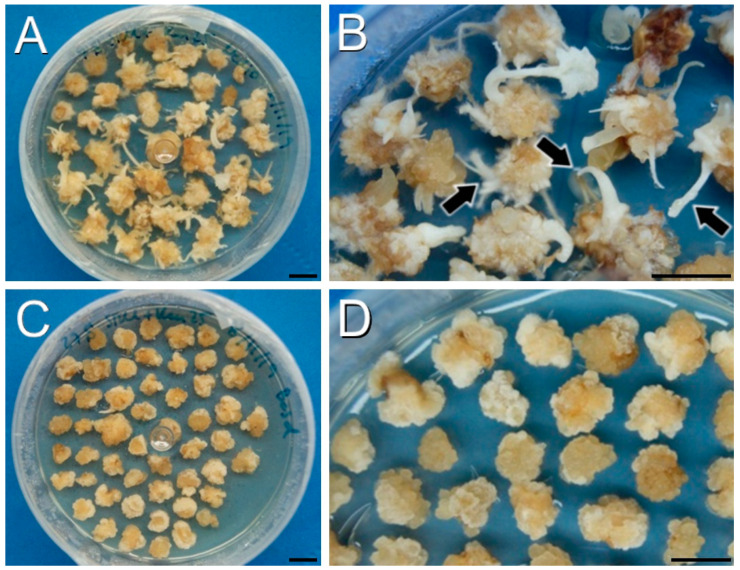
Callus production and growth using protocol B after one month. (**A**,**B**): DH4079 calli in SRM supplemented with 50 mg/L of kanamycin. Note the presence of adventitious organs (arrowheads in (**B**) in some of these calli. (**C**,**D**): DH12075 calli in SRM supplemented with 10 mg/L of BASTA. (**D**): Amplified image where the absence of regeneration is clear. Bars: 1 cm.

Protocol B was the only protocol that did not produce any plant (either transformed or not) per explant, while protocols Z and A were able to regenerate plants from both DH4079 and DH12075 lines (Table 1). Regeneration was seriously compromised with protocol B. Interestingly, the only difference between protocols B and A is the time point of the addition of the selective agent to the culture medium (Figure 1). In protocol A, the selective agent was added to SOM medium, whereas in protocol B it was added earlier, to SRM medium, and this appeared to be crucial for the final fate of explants. We used a kanamycin concentration of 50 mg/L since it was previously described to combine moderate rates of escapes and regeneration for successful *B. napus* transformation [22]. When 50 mg/L of kanamycin was used in SRM medium (for explants transformed with the PM-YC3.6-LTI6b plasmid), few adventitious roots and leaves were produced from the calli after one month of culture. After two months, all the calli from both DH4079 and DH12075 lines turned creamy or brown and arrested their growth, showing clear signs of necrosis in some cases (Figure 2A,B). No developed shoots were observed in any case. When SRM medium was supplemented with 10 mg/L of BASTA (for explants transformed with the YC3.6-bar plasmid), the regeneration capacity of the calli was completely inhibited. Neither adventitious organs nor shoots emerged from the DH4079 or DH12075 calli and after two months of culture, all callus tissue was dead (Figure 2C,D). Thus, the use of selective agents in these conditions prevented shoot regeneration before callus death.

**Figure 3:**
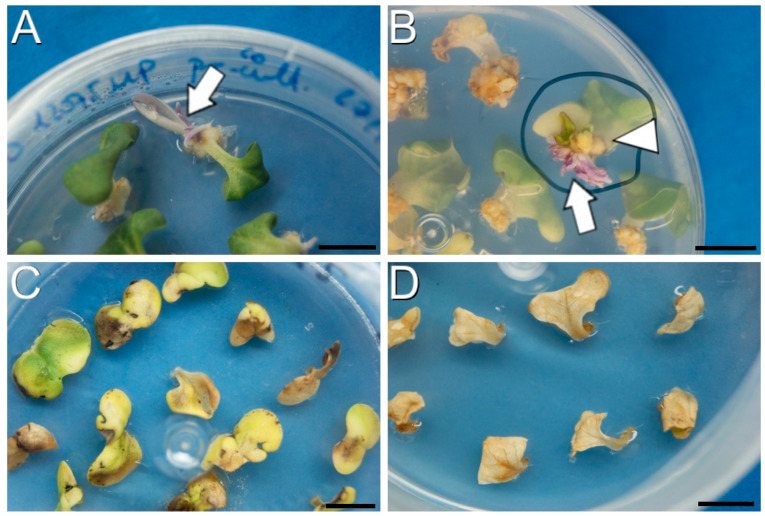
Callus production and growth using protocol Z. (**A**): Cotyledons after one month in selection medium (50 mg/L of kanamycin) showing a susceptible, purple shoot (arrow). (**B**): Cotyledons after two months in selection medium (50 mg/L kanamycin) showing resistant, green shoots (arrowhead) and susceptible, purple shoots (arrow). (**C**,**D**): Cotyledons after one (**C**) and two months (**D**) in selection medium with 5 mg/L of BASTA. Bars: 1 cm.

In protocol Z, cotyledons of the DH4079 and DH12075 lines were used as explants. With this protocol, the callus formation and growth were more limited than with protocol B, but clear differences in terms of regeneration were observed between explants exposed to different selective agents. After one month growing in selection medium with 50 mg/L of kanamycin, explants from the PM-YC3.6-LTI6b transformation developed the first shoots (Figure 3A) and after two months, resistant (green) and susceptible (purple) shoots were observed (Figure 3B). However, explants grown in selection medium supplemented with 5 mg/L of BASTA (for YC3.6-bar explants) did not develop any shoots. After one month of culture, visible signs of necrosis appeared in the explants (Figure 3C) and after two months, all cotyledon explants died (Figure 3D). Thus, the use of kanamycin allowed for plant regeneration from explants, whereas the use of BASTA promoted their death.

The conditions of use of the selective agent in culture media has been reported as one of the main factors affecting transformation efficiency [19]. Indeed, in the *B. napus* cv. Westar, an increase in selection conditions from 50 to 100 mg/L of kanamycin resulted in a reduction of non-transformed regenerated shoots (escape shoots), but the regeneration rate dropped down from 19% to 13% [22]. However, kanamycin concentration did not seem to be a problem in our case, since for protocol A, regenerants were obtained with this concentration in both protocols B and Z. Instead, the problem in protocol B seemed to be the time point of addition of the selective agent. A similar effect of kanamycin in plant regeneration has already been described in other species, such as grapevine [29], carrot [30] and cotton [31]. A similar scenario appears to occur with the use of BASTA in protocol B. However, BASTA was toxic in protocol Z. In general, herbicides have not been commonly used as selective agents as much as antibiotics due to the difficulty of establishing efficient concentrations which permit tissue regeneration and transformed plant selection [32], as it appears to occur in our *B. napus* explant transformations using BASTA in the selective medium. Such an inhibitory effect of BASTA in plant regeneration has also been described at even lower concentrations in watermelon [33] and peach [34]. Therefore, the addition of the selective agent (either kanamycin or BASTA) in the early steps of the protocol, when organogenesis is not yet initiated, results in an arrest of growth and organogenic differentiation, as well as the use of BASTA at both 5 (protocol Z) and 10 mg/L (protocol B).

### 2.2. The DH12075 Line, but Not DH4079, Can Be Genetically Transformed Using Protocol A

**Figure 4:**
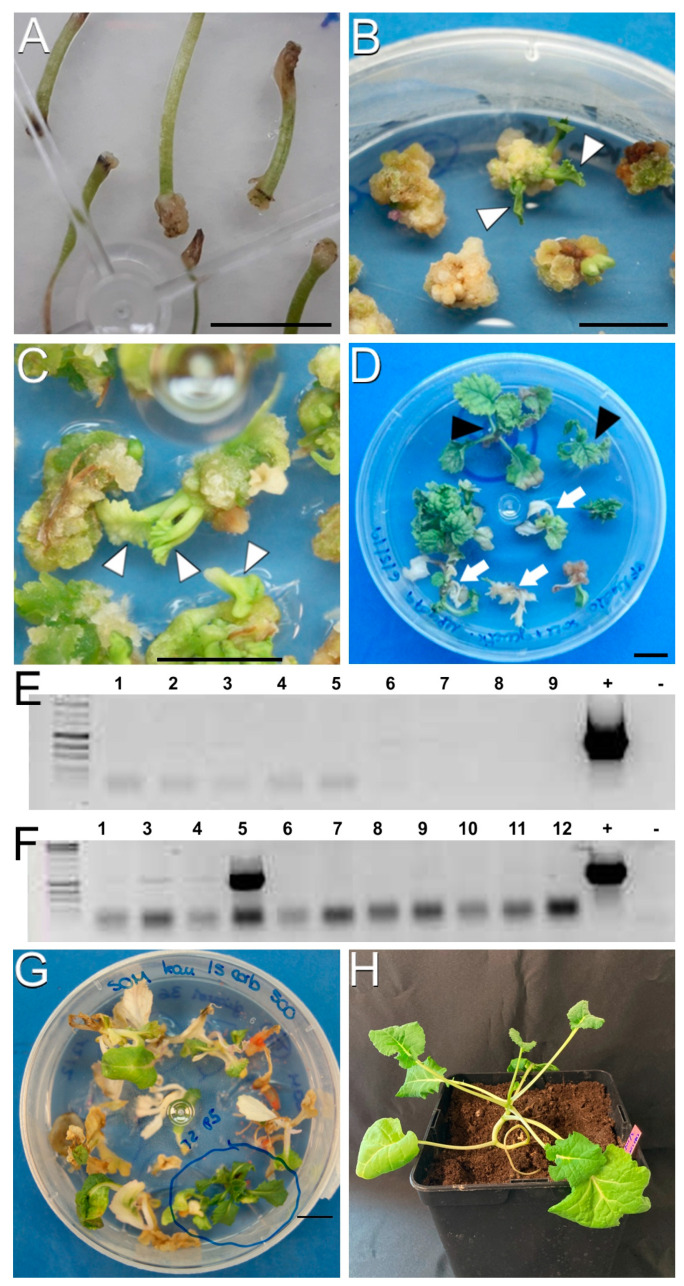
Callus production and plant regeneration using protocol A. (**A**): Explants after 2 weeks in CIM + carbenicillin. (**B**): Calli after 4 weeks in SRM + carbenicillin. Note the occurrence of some green shoots on the callus surface (arrowheads). (**C**): Shoots (arrowheads) emerging from calli after 6 weeks in SRM + carbenicillin. (**D**): Green, regenerated plantlets resistant to the selective agent (black arrowheads) and white, arrested shoots sensitive to the selective agent (white arrows) after three weeks in SOM + carbenicillin + kanamycin. (**E**,**F**): PCR genotyping of green, transformed plants of the DH4079 (**E**) and DH12075 lines (**F**), using YC3.6 Fw and GFP 5′ Rv primers. Plasmid DNA was used as PCR positive control (+) and water was used as negative control (-). Note the presence of a PCR-positive DNA sample in (**F**), lane 5. (**G**,**H**): Green regenerated transgenic in vitro (**G**) and acclimated (**H**) plant of the DH12075 line. Bars: 1 cm.

The protocol showing the highest plant-regeneration ratio was protocol A (Table 1 and Table 2), where inoculation consisted of the submersion of hypocotyl explants in the *Agrobacterium* suspension. With protocol A, calli were produced at the cuttings of hypocotyl explants after two weeks of culture (Figure 4A). Upon individualization of the calli, the first evidence of shoot formation was visible after approximately four weeks of culture (Figure 4B, arrowheads) and after six weeks, clearly visible, well-formed shoots were visible (Figure 4C, arrowheads).

Plants were produced from both DH4079 and DH12075 lines transformed with YC3.6-Bar, PM-YC3.6-LTI6b and pCRISPR. As opposed to the other protocols used, no differences were observed in terms of regeneration efficiency between the use of kanamycin and BASTA. After three weeks in SOM medium supplemented with carbenicillin and the corresponding selective agent, some plants turned whitish and stopped growing (Figure 4D, arrows), indicating sensitivity to the selective agent. Others were able to survive in the presence of the selective agent and kept growing green and vigorous, regenerating plantlets (Figure 4D, arrowheads). These results confirmed that it is possible to regenerate plantlets from *B. napus* DH4079 and DH12075 lines using protocol A, whose regeneration ability did not depend on the explant genotype or the type of selective agent used.

To assess whether regenerated green, growing plantlets incorporated the plasmid, they were genotyped by PCR with different primer pair combinations (see *Materials and methods*). None of the regenerated plantlets from the DH4079 line tested positive for PCR (Figure 4E), whereas few plantlets regenerated from the DH12075-excised explants were genotyped as positive (Figure 4F), being able to grow to fully regenerated transgenic plants (Figure 4G,H), which resulted in a calculated efficiency of 0.65% in terms of PCR-positive plants regenerated per explant. In all the transformation and regeneration events performed, there was a difference between the number of total and PCR-positive plants per explant, revealing the presence of *escapes* (Table 1), defined as plants resistant to selective agents but not transformed with the corresponding construct. This was particularly high in the case of the YC3.6-Bar experiments (2.48 plants per explant, but not a single PCR-positive plant). We speculate that this high occurrence of escapes could be due to not sufficiently restrictive concentrations of the corresponding selective agent, as also reported in other backgrounds [22]. Notwithstanding this, we were unable to identify any single transformed DH4079 regenerant, which confirms the extreme recalcitrance of this DH line. Together, these results show that the *B. napus* DH lines DH12075 and DH4079 exhibit a remarkably low transformation efficiency. In particular, the DH4079 line is recalcitrant to *Agrobacterium*-mediated transformation using the protocols described so far, including the modifications presented in this work.

Traditionally, the DH4079 background has been considered as recalcitrant for transformation [24]. Indeed, despite the enormous practical applicability that the development of DH4079 lines transformed with different genetic markers would have for the study of microspore embryogenesis, no DH4079 transgenic lines have been reported up to date. Out of the 1784 explants excised from DH4079 plants and transformed, we were not able to identify any single transgenic plant. This contrasts with the 13.4% of transformation efficiency previously reported for 62 DH4079 explants transformed and regenerated using the same protocol [24]. The reasons for such discrepancy are difficult to elucidate. Possible explanations could be the use of DH4079 plant material that was somehow different, or the residual presence of *A. tumefaciens* in the plant material used to analyze the efficiency of transformation. To avoid these potential problems, we are confident in using the highly embryogenic *B. napus* DH4079 line, since we routinely perform microspore cultures with microspores isolated from plants of this line [35] and confirm their high embryogenic response. Moreover, plant samples for PCR were excised from leaves of regenerated plants at the 2–3 true leaf stage upon plant acclimation, avoiding possible *Agrobacterium* dragging within the sample. We used the same protocol previously published by Maheshwari et al. [24] adapted to our experimental conditions, and obtained similar regeneration rates. Overall, we obtained with such protocol a transformation efficiency for the DH12075 line similar to that previously described for the same line [36]. Therefore, we strongly believe that our data (both the successful transformation of DH12075 and the unsuccessful transformation of DH4079) are robust and consistent. The remarkably different amounts of DH4079 explants transformed, cultured and analyzed in both cases (1784 in this work vs. 62 in Maheshwari et al. [24]) support this notion. Therefore, we postulate that the DH4079 line of *B. napus* is recalcitrant to *A. tumefaciens*-mediated genetic transformation.

### 2.3. The B. napus DH4079 Line Is Also Recalcitrant to Transient A. rhizogenes Transformation

**Figure 5:**
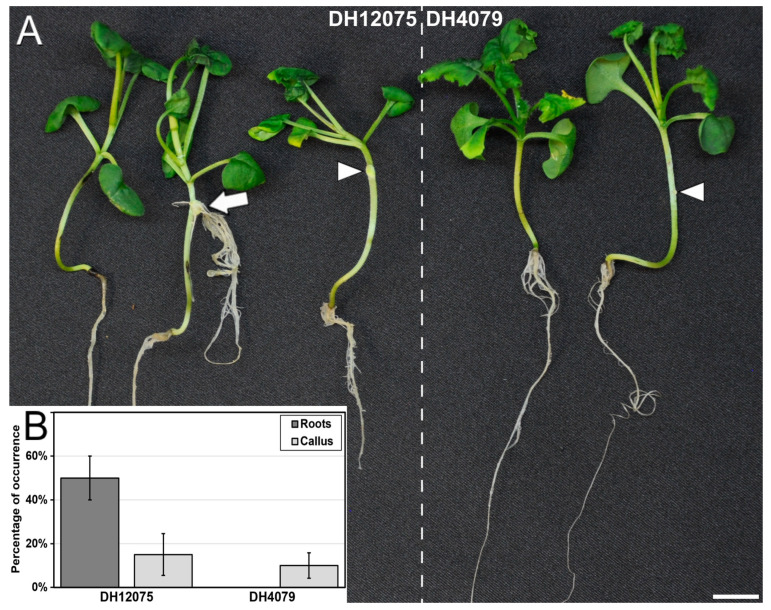
Response of *B. napus* DH12075 and DH4079 to transient *A. rhizogenes* infection. (**A**): The infected DH12075 plants (left) show in some cases no specific response, and in others callus formation (arrowhead) or formation of adventitious roots (arrow). In contrast, DH4079 plants (right) only show either no specific response in some plants or just callus formation (arrowhead) in others. (**B**): Comparison of the percentages of root and callus formation in DH12075 and DH4079. Bar: 1 cm.

Different methods of transient plant transformation have been tried in *B. napus*, including *A. tumefaciens* transformation of microspore-derived embryos [37] or microprojectile bombardment of isolated microspores combined with *A. tumefaciens* incubation [38], but not infection with *Agrobacterium rhizogenes*. *A. rhizogenes*, a gram-negative, soil-borne bacterium, naturally harbors large Ri plasmids that contain genes that favor infection of plant tissues and transference of their DNA into host plant cells [39]. However, as opposed to Ti plasmids from *A. tumefaciens*, Ri plasmids from *A. rhizogenes* induce the formation of hairy roots in the infected plant tissue [39]. Thus, genetic manipulation of these plasmids has allowed to establish protocols for transient plant transformation in different species, including *B. napus* [40]. We used this method to evaluate whether our *B. napus* lines show a response similar or different from that of *A. tumefaciens* stable transformation. We infected the hypocotyls of one-week-old entire plants of both DH12075 and DH4079 lines with *A. rhizogenes* and analyzed the occurrence of morphogenic processes as a consequence of infection after 3–4 weeks. In DH12075, we found plants with no specific response to infection, plants producing a callus at the site of wounding for infection, and plants developing large hairy roots at the site of infection, whereas DH4079 only presented plants with no specific response or with callus production (Figure 5A). The percentage of plants producing calli was similar for both lines, ranging between ~10–15% (Figure 5B), which may indicate a plant response to wounding rather than to bacterial infection. However, there was a remarkable difference in root production upon infection, which revolved around 50% for DH12075, but was null for DH4079 (Figure 5B). These results confirmed the recalcitrance of DH4079 to *Agrobacterium* transformation, not only with *A. tumefaciens* but also with *A. rhizogenes*.

A recent work reported the formation of hairy roots from both DH12075 and DH4079 when infected with a modified *A. tumefaciens* strain carrying a Ri hairy-root-inducing plasmid [41]. Hairy roots developed from 97% and 42% of the infected DH12075 and DH4079 seedlings, respectively, but transgenic plants were only possible to regenerate from transformed and cultured DH12075 roots [41]. This transformation approach using engineered *A. tumefaciens* with Ri plasmid is essentially different from the protocols used in the present work and may open an alternative approach to produce transgenic *B. napus* lines upon plant regeneration from transformed roots in different genotypes, including DH12075. However, it also failed in achieving stable transformation of DH4079. These observations, together with the work presented in this manuscript, support the notion that the DH4079 line is extremely recalcitrant to *Agrobacterium* transformation.

### 2.4. A Possible Relationship between Recalcitrance to Genetic Transformation and Doubled Haploidy?

Within the *Brassica* genus, efficiencies of stable transformation are remarkably variable, ranging between 0.59–1.56% for wucai (*B. campestris*) [42], 2.2–10.83% for Chinese cabbage (*B. rapa* ssp. *Pekinensis*) [43,44], 2.7–6.4% for broccoli (*B. oleracea* var. *italica*) [45], ~7% for *B. juncea* [46], 7–13.6% for *B. oleraceae* var *Botrytis* [47,48] and as high as 32.5–45% for cabbage (*B. oleracea* subsp. *capitata*), depending on the explant type (shoot tips and hypocotyls, respectively) [49]. As with *B. napus*, the efficiency reported by different works was highly dependent on the genetic background used. In commercial cultivars such as Oscar and RK7, an efficiency of up to 67% was reported [50]. In cv. Westar, a range of transformation efficiency of 7–33%, depending on in vitro conditions, was reported [22,50]. Interestingly, when fully homozygous DH plants derived from Westar were used in similar experimental conditions, the transformation efficiency dropped down to 0.3–3% [36]. Whether these differences are due to different specific experimental conditions, or to allele fixation derived from the process of chromosome doubling inherent to DH production [6], is not known. However, the transformation efficiency reported in our work for DH12075, a Westar-derived DH line, was 0.65%, which fits within the range of efficiencies reported for DH backgrounds [36]. This supports the hypothesis that recalcitrance to transformation could be influenced by the degree of gene fixation in a partially allogamous species such as *B. napus*.

Finally, it is also worth mentioning that DH4079, in addition to being recalcitrant to transformation, is one of the backgrounds most responsive to induction of microspore embryogenesis for DH production [11]. In parallel, some of the *B. napus* backgrounds where genetic transformation has been proven efficient or at least possible, such as Westar and DH12075 [41], are also known to show a very low or null response to induction of microspore embryogenesis [51,52]. Interestingly, it is also known that *Arabidopsis thaliana*, a model species for genetic studies where genetic transformation is very well developed and widely used for decades, is extremely recalcitrant to microspore embryogenesis, with no successful reports of induction of such morphogenic process published to date. The scenario in a tomato plant, another plant model species, is similar: transformation has been successfully achieved, but its extreme recalcitrance to microspore embryogenesis is widely acknowledged [53,54,55]. Thus, it is tempting to speculate about a possible inverse relationship between the ability for genetic transformation and the response to induction of microspore embryogenesis. This could be an interesting hypothesis to elucidate in future research.

## 3. Materials and Methods

### 3.1. Plant Material

Seedlings from two different *B. napus* DH lines were used as donors of explants: the DH4079 line, derived from cv. Topas [11], and the DH12075 line, derived from a cross between cvs. Westar × Cresor.

### 3.2. Constructs

**Figure 6:**
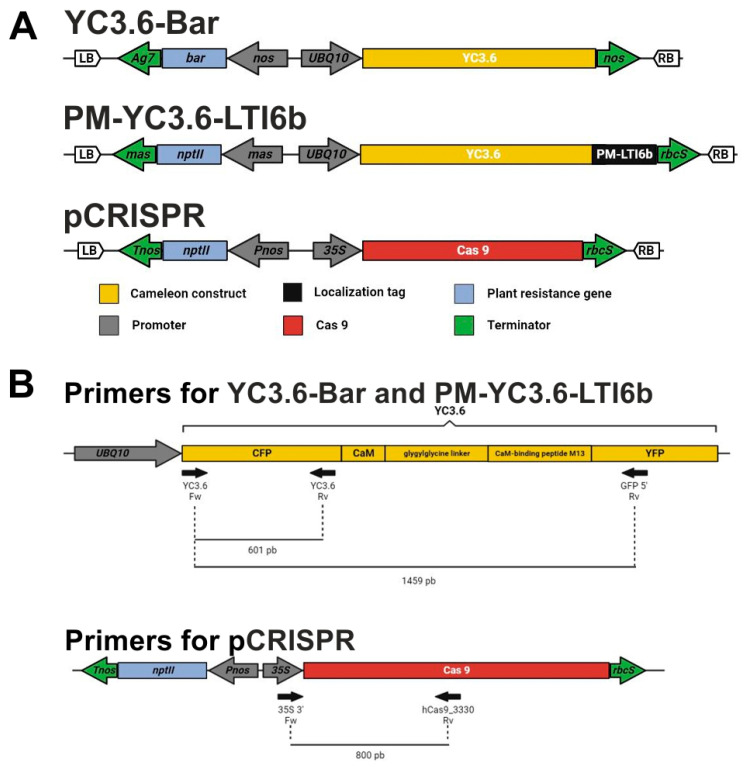
Diagrams of the vectors and primers used for *B. napus* transformation. (**A**). Vectors used for the optimization of *B. napus* transformation protocol: YC3.6-Bar, PM-YC3.6-LTI6b and CRISPR/Cas9 recombinant plasmid (pCRISPR). LB: Left Border; RB: Right Border; *Ag7*: *gene7*; *mas*: mannopine synthase; *UBQ10: UBIQUITIN10; nos:* nopalin synthase; *rbcS*: *ribulose bisphosphate carboxylase oxygenase small subunit*. (**B**). Scheme of the primers (black arrows) used to genotype the regenerated plants and their amplification products. The first scheme shows the primer combinations used for *cameleon* constructs (YC3.6-Bar and PM-YC3.6-LTI6b), and the second scheme shows the primers used for CRISPR/Cas9 constructs.

Three plasmids were used in this experiment (Figure 6A). On the one hand, we used YC3.6 and PM-YC3.6-LTI6b, two *cameleon* calcium sensor constructs that confer BASTA and kanamycin resistance, respectively, kindly provided by Dr. Jörg Kudla [56]. On the other hand, we used pCRISPR, a GoldenBraid-based construction for CRISPR/Cas9 genome editing [57] containing a CaMV 35S promoter, Cas9 CDS, and Nos Terminator cassette, assembled in the pDGB3-alpha1 binary plasmid containing a cassette for kanamycin for plant selection.

### 3.3. Management and Transformation of Escherichia coli and Agrobacterium Strains 

Chemically competent *Escherichia coli* One Shot™ ccdB Survival™ 2 T1R cells were prepared from cells growing under agitation and 37 °C in 50 mL Psi medium (Table 3) until the DO_600_ reached 0.4–0.5. Bacterial culture was cooled down and centrifuged (5 min, 5000× *g*, 4 °C). The pellet was resuspended in 20 mL of cold Transformation Buffer I (TBI; Table 3) and incubated on ice for 15 min. Next, the bacterial suspension was centrifuged (10 min, 2000× *g*, 4 °C) and the resulting pellet was resuspended in 2 mL of Transformation Buffer II (TBII; Table 3) and incubated in ice for 15 min. Finally, 50 µL aliquots were kept at −80 °C. For transformation, aliquots were thawed and 5 µL (~1 µg) of DNA was added. Tubes were incubated for 60 min on ice, 30 s at 42 °C and then on ice for 2 min. Next, 250 µL of autoclaved SOC medium (Table 3) were added and tubes were incubated at 37 °C for 1 h in a shaker at 225 rpm. From each tube, 25–100 µL were plated in commercial LB-Agar Miller medium (VWR Life Science) plates supplemented with the corresponding bacterial selective agents (Table 4) and incubated at 37 °C. Saturated *E. coli* liquid LB cultures were used to perform plasmid extractions with the NZYMiniprep kit (NZYtech).

*Agrobacterium tumefaciens* strain LBA4404 carrying the helper *vir* plasmid pAL4404 (ElectroMAX™ *A. tumefaciens* LBA4404 Cells, Invitrogen™, Waltham, MA, USA) was used for plant transformations. Electrocompetent *A. tumefaciens* cells were prepared by culturing a single colony in liquid YM medium (Table 3) for 2 days at 28–30 °C. One mL of culture was diluted 1:100 in liquid YM medium. The dilution was incubated at 28–30 °C in agitation until DO_600_ ranged 0.5–0.7. The *Agrobacterium* culture was centrifuged (15 min, 4 °C, 6000× *g*) and the pellet was washed with 30 mL of cold sterile MilliQ water. Centrifugation (same conditions) and washing was repeated twice. Finally, cells were resuspended in 1 mL of cold 10% (*v/v*) glycerol and 20 µL aliquots were stored at −80 °C. For transformation of *A. tumefaciens* LBA4404 competent cells, each aliquot was thawed, 1 µL (~200 ng) of plasmid was added and the mixture was pipetted into the electroporation cuvette. The mixture was electroporated at 2.0 kV, 200 Ω y 25 µF using an Electro Cell Manipulator 600 electroporator from BTX (Holliston, MA, USA). After electroporation, 1 mL of autoclaved liquid YM medium was added to the micro tube. The content was transferred to a 15 mL tube and incubated at 225 rpm and 28 °C for 3 h. Then, 50–100 µL were plated in solid YM (Table 3) medium with the corresponding plasmid-specific selective agents (Table 4). Plates were incubated at 28 °C for 2–3 days until colonies were observed. Preparation of *Agrobacterium rhizogenes* strain 15834 competent cells and transformation with pCRISPR by electroporation was performed as described above for *A. tumefaciens*, but using MGL liquid medium (Table 3).

### 3.4. Explant Stable Transformation and Plant Regeneration

Three *A. tumefaciens* stable transformation protocols were assayed (Figure 1), two (B and A) based on [24] and one (protocol Z) based on [21]. For all protocols, bacterial inoculums were prepared by centrifuging (5975× *g*, 10 h, 4 °C) liquid cultures grown at 28 °C in liquid YM medium (Table 3) supplemented with 50 mg/L rifampicin and appropriate bacterial selective agents (Table 4) until reaching an approximate OD_600_ = 1.0. The bacterial pellets were resuspended in liquid MS medium with 1% sucrose until reaching a OD_600_ ranging 0.15–0.2, and 100 µM acetosyringone was added. To produce the explant-donor seedlings, *B. napus* seeds of the DH4079 and DH12075 lines were surface-sterilized for 15 min in a 70% ethanol +0.1% Triton X-100 solution, followed by 15 min in 10% bleach solution and three rinses with distilled water. Seeds were then germinated in Germination Medium (GM; Table 5).

In protocols A and B, hypocotyl explants of 0.5–1 cm were excised from 7-day-old seedlings and pre-cultured in Callus Induction Medium (CIM; Table 5) for 5 days. After 3–5 days, explants were inoculated with the corresponding *A. tumefaciens* culture. Pre-cultured explants were submerged in the bacterial suspension, blotted on sterile paper and transferred to CIM medium for 72 h at 25 °C under reduced light conditions by covering the culture with a piece of white paper to reduce light intensity to dim light. After co-cultivation with *A. tumefaciens,* explants were transferred to CIM medium supplemented with 500 mg/L of carbenicillin and cultured for 2 weeks at 25 °C under dim light. The regenerated calli were cut into pieces and cultured for 4–6 weeks in Shoot Regeneration Medium (SRM; Table 5) for protocol A, and for protocol B in SRM medium supplemented with 50 mg/L of kanamycin as plant selection agent for PM-YC3.6-LTI6b transformation and 10 mg/L of BASTA (amonium glufosinate) for YC3.6-Bar. The developing shoot buds were transferred to Shoot Outgrowth Medium (SOM; Table 5) supplemented with plant selection agents as described above. Shoots longer than 2 cm were transferred to Root Regeneration Medium (RRM; Table 5) supplemented with plant selection agents as described above.

For protocol Z, cotyledon explants were obtained by cutting the petiole base from 5- to 7-day-old seedlings. Excised cotyledons were inoculated by dipping the petiole base for 3–5 s in liquid MS + V medium + 1% sucrose and the *A. tumefaciens* preparation (OD_600_ = 0.2 supplemented with 100 µM acetosyringone). Then, explants were inoculated by inserting 1–2 mm of the petiole base in solid Co-Cultivation Medium (CCM; Table 5). Plates were kept at 25 °C for 72 h under dim light. Then, explants were transferred to Selection Medium (SM; Table 5) supplemented with plant selection agents (15 mg/L of kanamycin for PM-YC3.6-LTI6b and CRISPRp transformations and 5 mg/L of BASTA for YC3.6-Bar) for at least four weeks, subculturing them to fresh SM every week. The regenerating shoots produced were transferred to Rooting Medium (RM; Table 5) supplemented with plant selection agents as described above.

For genotyping of regenerated plants, when putative transgenic plants developed 2–3 true leaves, 1 cm^2^ pieces of leaf tissue were excised and DNA was extracted on TNES extraction buffer (100 mM Tris Buffer, 100 mM EDTA, 250 mM NaCl, pH 8) and precipitated with 1 volume of isopropanol at room temperature for 2 min. The pellet was resuspended in 50 µL MilliQ water. The primer pairs used to genotype regenerated plants from transformations with PM-YC3.6-LTI6b, YC3.6-Bar and pCRISPR are shown in Figure 6B, and their respective sequences in Table 6. All PCRs were performed by adding 1 µM primers to the Mix MZY Taq II 2x Green Master Mix (NZYtech, Lisbon, Portugal), with annealing temperature of 59 °C and extension time of 40 s.

### 3.5. A. rhizogenes-Mediated Transient Transformation

For transient hairy root transformation of *B. napus* with *Agrobacterium rhizogenes*, the protocol was adapted from Ron et al. [26]. Briefly, 3 mL of saturated *A. rhizogenes* culture on MGL liquid medium supplemented with kanamycin (50 mg/mL) was centrifuged at 5000× *g*, 4 °C for 15 min, and the obtained pellet was resuspended in 200 µL of fresh MGL liquid medium. The resulting concentrated bacterial suspension was used to infect 10-day-old *B. napus* seedlings sowed and grown as described above and cultured on solid MS media (0.8% plant agar) under sterile conditions. For *A. rhizogenes* infection, a sterile needle tip was impregnated with the concentrated bacterial suspension, and two soft, little wounds were made along the stem of each seedling. Plantlets were then cultured in vitro for 3–4 additional weeks under the same conditions, and the numbers of roots produced were then registered.

### 3.6. Statistical Analysis

Plant regeneration data were analyzed using the StatGraphics software, v18.1.13. The Kruskal–Wallis test with *p* ≤ 0.05 was used to detect statistically significant differences in plant regeneration and transformation rates.

## Figures and Tables

**Table 1:** Summary of the results of DH4079 and SH12075 *B. napus* transformation with the B, Z and A protocols.

Genotype	Protocol	Plasmid	Resistance	Explants	Plants/Explant	Percentage of Positive Plants
4079	B	YC3.6-Bar	BASTA	296	0.00	0%
4079	B	PM-YC3.6-LTI6b	Kanamycin	266	0.00	0%
12075	B	YC3.6-Bar	BASTA	285	0.00	0%
12075	B	PM-YC3.6-LTI6b	Kanamycin	403	0.00	0%
4079	Z	YC3.6-Bar	BASTA	138	0.00	0%
4079	Z	PM-YC3.6-LTI6b	Kanamycin	143	0.02	0%
12075	Z	YC3.6-Bar	BASTA	98	0.00	0%
12075	Z	PM-YC3.6-LTI6b	Kanamycin	138	0.46	0%
4079	A	YC3.6-Bar	BASTA	358	2.48	0%
4079	A	PM-YC3.6-LTI6b	Kanamycin	361	0.64	0%
4079	A	CRISPR	Kanamycin	222	0.12	0%
12075	A	CRISPR	Kanamycin	153	0.19	3.4%

**Table 2:** Effect of the protocols on the regeneration capacity of plants. Different letters indicate statistically significant differences according to LSD test (*p* ≤ 0.05).

Protocol	Plants/Explant
B	0.00 ± 0.00 ^b^
Z	0.12 ± 0.06 ^ab^
A	1.10 ± 0.12 ^a^

**Table 3:** Composition of the different culture media used for management and transformation of bacterial strains. TBI: Transformation Buffer I; TBII: Transformation Buffer II.

	Psi	TBI	TBII	SOC	Liquid YM	Solid YM	MGL
Potassium acetate (mM)		30					
RbCl_2_ (mM)		100	10				
CaCl_2_·2H_2_O (mM)		10	75				
MnCl_2_·4H_2_O (mM)		50					
MOPS (mM)			10				
MgCl_2_ (mM)	52.63			10			
NaCl (mM)				10	1.7	1.7	85.5
KCl (mM)				2.5			
MgSO_4_ (mM)				10			
MgSO_4_·7H_2_O (mM)					0.8	0.8	0.4
Glucose (mM)				20			
K_2_HPO_4_·3H_2_O (mM)					2.2	2.2	1.4
Tryptone (%)	2			2			0.5
Yeast extract (%)	0.5			0.5	0.04	0.04	0.25
Mannitol (%)					1	1	0.5
Glycerol (% *v/v*)		15	15				
L-glutamic acid (g/L)							1
Biotin (mg/L)							1
Bacteriological agar (%)						1	
Rifampicin (mg/L)						50	
pH	7.6	5.8	6.5	7	7	7	7

**Table 4:** Plasmid selection agents used for bacterial transformation.

	PM-YC3.6-LTI6b	YC3.6-Bar	CRISPRp
Kanamycin		50 mg/L	50 mg/L
Spectinomycin	100 mg/L		
Streptomycin	100 mg/L		

**Table 5:** Composition of the different culture media used for explant transformation and plant regeneration. 2,4-D: 2,4-dichlorophenoxyacetic acid; BAP: 6-benzylaminopurine; CCM: Co-Cultivation Medium; CIM: Callus Induction Medium; GM: germination medium; MS + V: Murashige and Skoog basal medium including vitamins (Duchefa Biochemie); RRM: Root Regeneration Medium; SM: Selection medium; SOM: Shoot Outgrowth Medium; SRM: Shoot Regeneration Medium.

	GM	CIM	SRM	SOM	RRM	CCM	SM	RM
MS + V (g/L)	4.6	4.6	4.6	4.6	2.3	4.6	4.6	2.3
MES (g/L)		2.5	2.5	2.5	2.5			
Myo-inositol (g/L)		1	1	1	1			
AgNO_3_ (mg/L)		5	5					
2,4-D (mg/L)		1						
BAP (mg/L)			5	0.05		2	2	
Sucrose (g/L)	20	30	30	30	10	30	30	10
Plant agar (g/L)	8	8	8	8	8	8	8	8
Carbenicillin (mg/L)			500	500	100		500	500
pH	5.8	5.8	5.8	5.8	5.8	5.8	5.8	5.8

**Table 6:** Oligonucleotides used as primers (Fw: Forward primer; Rv: Reverse primer).

Oligonucleotide	Sequence (5′ to 3′)
35S 3′ Fw	GATGACGCACAATCCCACTATCC
hCas9_3330 Rv	GCAGAATGGCGTCTGACAGG
YC3.6 Fw	TAAACGGCCACAGGTTCAGC
YC3.6 Rv	CGATCACATGGTCCTGCTGGA
GFP5′ Rv	GCGACGTAAACGGCCACAAGTTCAG

## Data Availability

All data supporting the findings of this study are available within the paper.
